# Optimal Digital Implementation of Fractional-Order Models in a Microcontroller

**DOI:** 10.3390/e22030366

**Published:** 2020-03-23

**Authors:** Mariusz Matusiak, Marcin Bąkała, Rafał Wojciechowski

**Affiliations:** Institute of Applied Computer Science, Łódź University of Technology, ul. Stefanowskiego 18/22, 90-924 Lodz, Poland; mmatusiak@iis.p.lodz.pl (M.M.); rafal.wojciechowski@p.lodz.pl (R.W.)

**Keywords:** fractional calculus, Grünwald–Letnikov differintegral, BLDC motor model, microcontroller implementation

## Abstract

The growing number of operations in implementations of the non-local fractional differentiation operator is cumbersome for real applications with strict performance and memory storage requirements. This demands use of one of the available approximation methods. In this paper, the analysis of the classic integer- (IO) and fractional-order (FO) models of the brushless DC (BLDC) micromotor mounted on a steel rotating arms, and next, the discretization and efficient implementation of the models in a microcontroller (MCU) is performed. Two different methods for the FO model are examined, including the approximation of the fractional-order operator $s^{\nu }$ (ν∈R) using the Oustaloup Recursive filter and the numerical evaluation of the fractional differintegral operator based on the Grünwald–Letnikov definition and Short Memory Principle. The models are verified against the results of several experiments conducted on an ARM Cortex-M7-based STM32F746ZG unit. Additionally, some software optimization techniques for the Cortex-M microcontroller family are discussed. The described steps are universal and can also be easily adapted to any other microcontroller. The values for integral absolute error (IAE) and integral square error (ISE) performance indices, calculated on the basis of simulations performed in MATLAB, are used to evaluate accuracy.

## 1. Introduction

Optimal solutions for implementing models of plants are of great interest to industry, since they enable the extension of the computer-aided simulations performed in computation software such as MATLAB/Simulink. In numerous control systems, it is essential that the process of tuning the controller on the basis of measurements of system output involves as few costly plant identification iterations as possible. A common solution involves synthesizing the plant model from the measured characteristics and implementing it in a dedicated software environment or microprocessor-based hardware platform. Significant difficulties arise when the models are described by fractional-order calculus (FOC) [1,2,3]. Much research on fractional-order control systems uses computational software for analysis and simulations. A noticeably smaller proportion addresses the problem of digital implementation of FOC equations on real devices, not only theoretically, but also practically [4,5,6,7,8]. In contrast to the well-known bounded numerical approximations of a classic integer-order derivative, such as backward or central differences, the problem arises of a constantly increasing number of discrete convolution operations over time. In order to reduce the negative impact of this issue, numerous approximation methods have been proposed [9,10,11,12], divided between the time-domain and frequency-domain. In the time domain, limited memory-based approaches are the most popular, including the Short Memory Principle (SMP) algorithm introduced by Igor Podlubny [3]. In the frequency domain, a selected range of Bode characteristics G(ω) can be approximated using the well-known Oustaloup Recursive filter algorithm (ORA) [10] or modifications thereof. Hardware implementation of fractional order models in control engineering is of great interest for the purposes of offline controller tuning and testing in already developed industrial control systems, which must not be affected in any way. An accurate, equivalent mathematical model realized as a dedicated hardware platform is usually desired. It can be very useful in such cases to use a microcontroller (MCU) as the target plant, with the output signal calculated on the basis of a transfer function of its model. Alternatively, one can consider designing an equivalent, time-continuous fractance circuit. In our research, we focus on the former approach, touching on the problem of discretization of the fractional-order model and combining approximation methods with universal optimization programming techniques to improve performance, reduce computation time, and limit the size of the occupied microcontroller memory with a negligible impact on accuracy. As a rule of a thumb, optimal implementation allows a higher order *N* of approximation formulas, producing more complex but accurate equations, which can be computed during the same constant sampling period. The presented example of microcontroller implementation is an essential part of the testing hardware platform, which is designed for the purpose of developing a sophisticated variable fractional-order PID (VFOPID) controller to be used in a closed-loop control system with multiple brushless DC (BLDC) motors. The paper is arranged as follows: In Section 2, the proposed testing platform and plant models are described. A description of the MCUs selected for the experiments is also provided. In Section 3 and Section 5 approximation and discretization techniques, useful for the implementation of the models on the target platform, are discussed. Two approaches are considered: approximation with an ORA and numerical evaluation of the fractional differential equation using a truncated Grünwald–Letnikov (GL) definition. Some remarks related to implementation are given in Section 4. Conclusions are given in the final Section 6.

## 2. Plant Models

**Figure 1:**
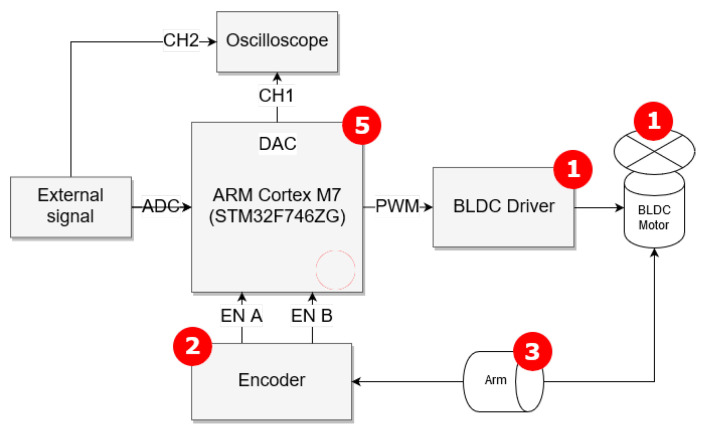
Block scheme of the testing hardware platform (1-BLDC micromotor, 2-high-precision encoder, 3-adjustable arm, 5-controller).

**Figure 2:**
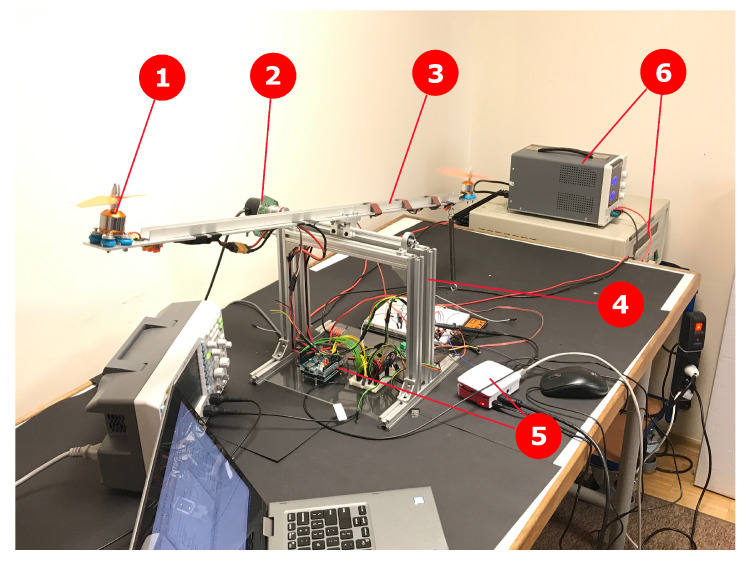
UAV arm testing platform with the BLDC motor and microcontroller (1-BLDC micromotor, 2-high-precision encoder, 3-adjustable arm, 4-rigid frame, 5-controllers, 6-power supply).

The closed-loop control system of an unmanned aerial vehicle (UAV) quadcopter arm, presented in Figure 1 and Figure 2, consists of a hardware platform with two micro BLDC motors, an encoder and a controller for modeling and designing an accurate control law for a dedicated fractional-order PID (FOPID) controller [3]. For small angles we treated the plant as a black box and provided classic, first and second integer-order Küpfmüller models, further enhanced by a model described by the fractional-order transfer function (FOTF). Matching the response of simple fractional order model approximation with the original data exceeded first and second-order Küpfmüller models. However, several assumptions and specific implementation techniques had to be considered, which will be described in the sections that follow. The transfer functions of the models, prepared in MATLAB R2017b computation software using PID Tuner applet, Optimization Toolbox and FOMCON [13,14] are:

1. First-order plus dead time (FOPDT)
  (1) $$G_{FOPDT}\left(s\right)=\frac{K_{P}}{\left(1+T_{P}s\right)}e^{\left(-T_{D}s\right)}=\frac{1}{\left(1+0.4934s\right)}e^{-1.2279s}$$
  where $K_{P}$ denotes the gain of the model, $T_{P}$ is a time constant and $T_{D}$ is the delay of the plant.
2. Second-order plus dead time (SOPDT)
  (2) $$G_{SOPDT}\left(s\right)=\frac{K_{P}}{{\left(1+T_{P}s\right)}^{2}}e^{\left(-T_{D}s\right)}=\frac{1}{{\left(1+0.319s\right)}^{2}}e^{-1.064s}$$
3. Non-integer-order plus dead time (NIOPDT)
  (3) $$\begin{array}{c} G_{NIOPDT}\left(s\right)=\frac{K_{P}}{a_{n}s^{v_{n}}+a_{n-1}s^{v_{n-1}}+\ldots +a_{0}s^{v_{0}}}e^{\left(-T_{D}s\right)}=\\ \frac{1}{\left(0.18234s^{1.9909}+0.65536s^{0.98319}+0.9992\right)}e^{-1s} \end{array}$$
  where $a_{n}\ldots a_{0}$ denote constants and $\nu_{n}\ldots \nu_{0}$ values of fractional orders.

For the purpose of experiments on a real hardware platform, the ARM Cortex-M7 core-based 32-bit microcontroller from the STM32 High Performance series, model STM32F746ZG [15] was used. The following configuration was being set up during the main program initialization routine:

- maximum value of the main clock frequency $f_{CPU}=216$ MHz,
- analog-to-digital converter (ADC) synchronized with the internal timer interrupt routine (ISR) to sample the input signal on an ADC pin at $f_{ADC}=f_{s}=1$ kHz,
- number of ADC domain clock cycles required for a single ADC conversion $c_{ADC}=15$ providing the best possible accuracy of 12-bit resolution. Time of conversion $t_{conv\;}\approx 10\;\mu s$,
- single-precision hardware floating-point unit (FPU) and compiler warnings on automatic double-precision promotion enabled (software-simulated support for double-precision arithmetic),
- 60 Hz PWM output signal with adjustable duty cycle for driving the 11VDC-supplied micro-BLDC driver.

## 3. Discretization

As mentioned in the previous chapter, integer-order models can be easily implemented on any microcontroller, in the form of digital finite (FIR) or infinite impulse response (IIR) filters [16,17]. A significantly better performance, due to the lower number of operations (feedforward and feedback taps), can be achieved with models implemented as IIR filters, which is important specifically for real-time calculations performed on a microcontroller at the cost of potential breakdown of stability. Discretization of the transfer functions (1) and (2) was performed using two well-known methods, the zero-order hold and bilinear transform (Tustin’s), at a sampling frequency of fs=1 kHz. The zero-order hold was selected for the best matching with the original characteristics.

**Figure 3:**
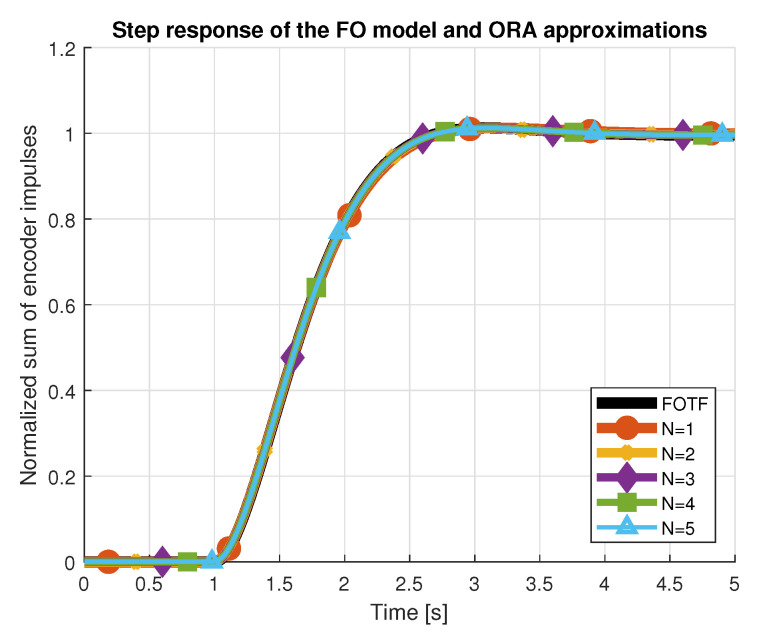
Oustaloup approximations of order *N* for fractional-order models. Step responses.

**Figure 4:**
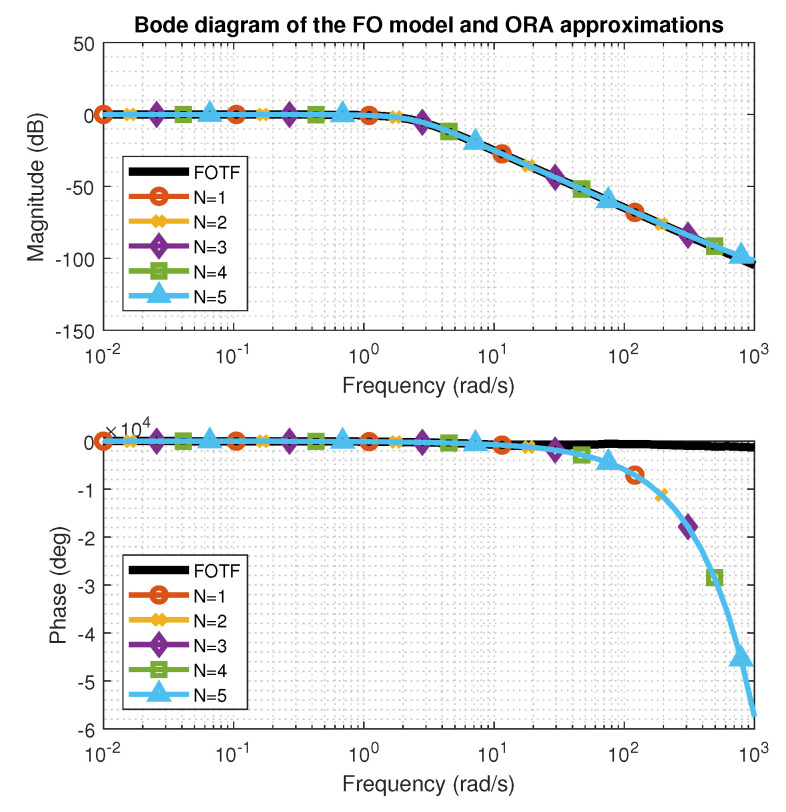
Oustaloup approximations of order *N* for fractional-order models. Bode diagram.

1. Discrete first-order plus dead time (DFOPDT)
  (4) $$H_{FOPDT}\left(z\right)=\frac{b_{0}+b_{1}z^{-1}}{1-a_{1}z^{-1}}z^{-T_{D}}$$
  where $b_{i}$ and $a_{j}$ are numerator and denominator coefficients, respectively, and $T_{D}$ is the number of delay input samples at a given sample rate $f_{s}$. The exact values of the coefficients are presented in Table 1.
2. Discrete second-order plus dead time (DSOPDT)
  (5) $$H_{SOPDT}\left(z\right)=\frac{b_{0}+b_{1}z^{-1}+b_{2}z^{-2}}{1-a_{1}z^{-1}-a_{2}z^{-2}}z^{-T_{D}}$$
3. Discrete non-integer-order plus dead time (DNIOPDT)
  Discretization of the non integer-order transfer function (3) was performed in three consecutive steps. First, approximation of the transfer function in the frequency domain was obtained by applying Oustaloup’s Recursive filter algorithm (ORA) [10,18], approximating the complex variable *s* of the fractional order 0 < ν < 1, using the following formula:
  (6) $$\begin{array}{c} s^{v}\;\approx K\prod_{k=-N}^{N}\frac{s+{\omega^{\prime }}_{k}}{s+\omega_{k}}=\omega_{h}^{v}\frac{\left(s-{\omega^{\prime }}_{-N}\right)\left(s-{\omega^{\prime }}_{-N+1}\right)\ldots \left(s-{\omega^{\prime }}_{N}\right)}{\left(s-\omega_{-N}\right)\left(s-\omega_{-N+1}\right)\ldots \left(s-\omega_{N}\right)} \end{array}$$
  where $\left[\omega_{b},\omega_{h}\right]$ denotes the frequency range of the approximation, *N* is the order and ${\omega^{\prime }}_{k}\;=\;\omega_{b}{\left(\frac{\omega_{h}}{\omega_{b}}\right)}^{\frac{k+N+0.5-0.5v}{2N+1}}\;,\;\omega_{k}=\omega_{b}{\left(\frac{\omega_{h}}{\omega_{b}}\right)}^{\frac{k+N+0.5+0.5v}{2N+1}}$. As a result, function (6) generates (2N+1) poles and zeros in total. If ν>1 then $s^{\nu }$ is first replaced with $s^{\left(n+u\right)}=s^{n}s^{u}$, where *n* is an integer number and *u* is a fractional part, approximated by the algorithm. Different values for the approximation order *N* were tested over the selected frequency range $\omega \in \left[10^{-4},\;10^{3}\right]\frac{rad}{s}.$ Step response and Bode characteristics for N∈[1,5] are presented in Figure 3 and Figure 4. It is noticeable that all values of the order *N* provide satisfactory approximations of the initial fractional order transfer function. Nevertheless, we proceeded with approximation orders N≥3. Since the approximation polynomials had over 14 zeros and poles, in the second step, a reduction was performed using the balancing reduction technique [19], available in MATLAB as *balred* method. Minimization of the cost functions for a new reduced-order plant model of a fixed *balred* order M=3,M∈N revealed that of several ORA filters, approximation of the order N=3 ensured the best match between both characteristics.
  (7) $$\begin{array}{c} G_{NIOPDT}\left(s\right)\approx \frac{0.00142\;s^{3}-\;0.01047\;s^{2}+5.75346\;s\;+\;2.01679}{s^{3}+\;4.10156\;s^{2}+\;7.13415s\;+\;2.01552}e^{-1s} \end{array}$$

**Figure 5:**
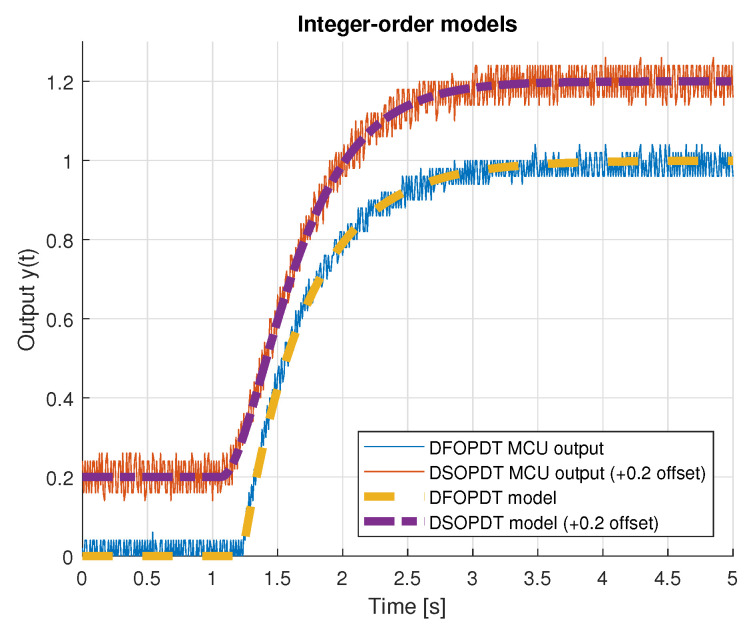
Measured microcontroller outputs with implemented integer-order (1st and 2nd) models.

**Figure 6:**
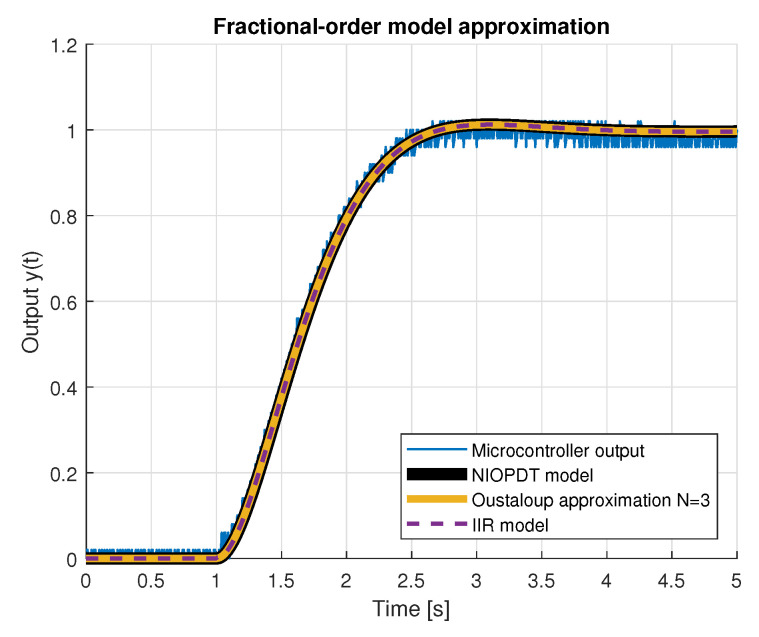
Measured microcontroller outputs with fractional-order (ORA) models.

Figure 5 and Figure 6 present unit step responses of the implemented platform integer- and fractional-order models, the latter approximated using the recursive Oustaloup filter method. Discrete 2nd-order model characteristics are presented with +0.2 offset to improve visibility.

## 4. Implementation Difficulties

It is important to stress two general points related to implementation. When discrete transfer functions (see Table 1) are obtained in MATLAB, one should be aware of the default Short Fixed Decimal display format, which rounds the numbers to four decimal places. Double precision representation of coefficients $b_{i}$, $a_{j}$ can appear in the Variables explorer or after the activation of the long format display mode using the MATLAB routine: *format long*. This is necessary to avoid the model from losing stability caused by the implementation of truncated values for the coefficients. It is first necessary to determine the desired precision of the floating-/fixed-point number representation in the microcontroller software and the presence of the hardware floating-point unit, as these factors have a great impact the performance of the algorithm. The transfer functions of the proposed IIR filters were transformed into difference equations and implemented on an STM32F746ZG microcontroller in C programming language. To preserve the asymptotic stability of the designed models, in the case of fractional-order approximation the calculations had to be performed using software simulated double-precision arithmetic. This had a significant impact on performance, increasing the required number of CPU cycles from $c_{avg,sp}=2650$ to $c_{avg,dp}=8500$ (320%). The alternative approach involved the evaluation of the FOTF in the time domain, as will be described in the next section.

## 5. Time-Domain Approach Using the Grünwald–Letnikov Differintegral Operator and SMP

To compare the efficiency and accuracy of the Oustaloup approximation, we considered a fractional-order differential equation evaluated in the time-domain on the basis of the implementation of the truncated Grünwald–Letnikov differintegral operator [20]. This technique is known as the Short Memory Principle and restricts the boundaries of the operations to the most recent $N_{l}$ samples. The principle is applied usually to numerical evaluation but has been also proposed for Riemann–Liouville and Caputo definitions [21]. Several different maximum memory lengths were examined $N_{l}=\left\{N_{0}=\frac{t_{sim}-t_{0}}{h},\;\frac{N_{0}}{2},\frac{N_{0}}{5},\frac{N_{0}}{10},\frac{N_{0}}{20},\frac{N_{0}}{50},\frac{N_{0}}{100}\right\}$ where $N_{0}$ denotes the total number of samples from the start of the simulation $t_{0}=0$ s, and was used as a reference value. The time responses of the plant were obtained using a modified formula [22]:

(8) $$\begin{array}{c} y\left(kh\right)=\frac{1}{\sum_{i=0}^{n}\frac{a_{i}}{h^{\nu_{i}}\;}}\left[u\left(kh\right)-\sum_{i=0}^{n}\frac{a_{i}}{h^{\nu_{i}}}\sum_{j=1}^{N_{k}}w_{j}^{\nu_{i}}y\left(kh-jh\right)\right] \end{array}$$

where $w_{j}^{\nu_{i}}$ denotes the Newton binomial weights in the GL definition:

(9) $$w_{j}^{\nu_{i}}=\left\{\begin{array}{cc} 1 & \mathrm{for}\;j=0\\ w_{j-1}^{\nu_{i}}\left(1-\frac{1+\nu_{i}}{j}\right) & \mathrm{for}\;j=1,2,\ldots \end{array}\right.$$

and $N_{k}$ is the number of previously processed samples:

(10) $$N_{k}=\left\{\begin{array}{cc} k & \mathrm{for}\;k\leq N_{l}\\ N_{l} & \mathrm{for}\;k>N_{l} \end{array}\right.,l\in \left[0,6\right]$$

**Figure 7:**
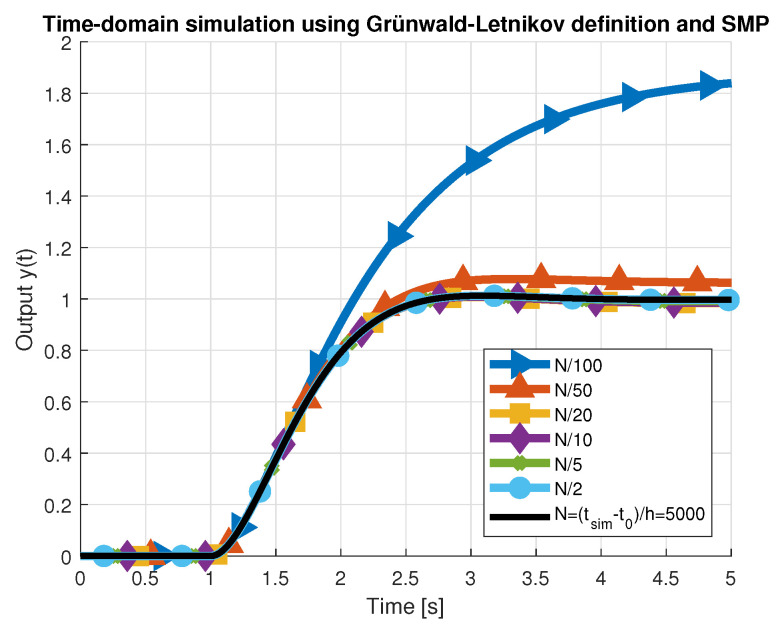
Heaviside step response evaluated by the GL method and different memory lengths *N*.

Figure 7 presents simulated characteristics for all values of $N_{l}$ and Table 2 below shows the corresponding performance indices, including those obtained for ORA. As can be seen, reducing the number of past samples below $N_{4}=\frac{N_{0}}{20}$ (green curve) generates considerable error, which can increase even more for plants characterized by longer transient states. Therefore, SMP lengths of $N_{5}$, $N_{6}$ were not considered for further analysis. Reduced-order approximations obtained using the ORA algorithm provided better results than nearly all SMP-based approximations. However, the value of the maximum absolute percentage error (MaxAPE) was usually higher, due to the deviation between the step response characteristics at the beginning of the transient state, near zero. This error dropped rapidly for k→∞. Only for the memory lengths of SMP $N\geq N_{1}$ were the step response characteristics (red curve) more accurate and similar to the initial step response of the fractional-order transfer function (3). The number of CPU cycles required to evaluate the output signal was measured using the Data Watchpoint and Trace unit of the microcontroller [23], by computing the difference between the values in CYCCNT register, read in two separate sections of the program. Several different software optimization techniques were applied to the algorithm in each iteration.

### 5.1. Initial Implementation- Look-up Tables, Shifted Input/Output Samples

In this step, arrays of lengths $N_{l}$ were dynamically allocated to storing double-precision input and output values and $w_{j}^{i}$ weight coefficients for each differintegral in the transfer function (3). $w_{j}^{i}$ coefficients were precomputed at the program initialization (look-up table). During each analog-digital conversion, a new input sample was added to the end of the input array and the values in the input and output arrays were shifted left when the limit $N_{l}$ was reached. Moreover, in the developed functions, only pointers to structures and arrays were accepted as parameters, to reduce the amount of memory occupied by the stack.

### 5.2. Replacing Arrays with Ring Buffers

Instead of shifting the values in the input/output arrays, a structure called a ring (circular) buffer was used. This involves defining a moving writing pointer (e.g., *inWrIdx*) for each of the arrays. When the buffer limit is reached (*inWrIdx* = $N_{l}$), the value is reset to point to the beginning of the buffer. The input value indicated by the pointer is always the most recent, whereas *inWrIdx*+1 (or 0 if *inWrIdx* = $N_{l}-1$) points to the oldest sample. For models with delay, an additional delay buffer with two pointers for writing and reading is initialized. Further optimization can be achieved when the array lengths are powers of two. At the cost of higher memory consumption, the conditional operator for checking the limit $N_{l}$ is replaced with a much faster bitwise multiplication of the pointer by $N_{l}$.

### 5.3. Enabling Optimization Flags

For the purposes of debugging and results verification, the program was initially built without any optimization by the GCC compiler (–O0 flag). Using the values $x=\left[1,3\right]$ with the flag –Ox may reduce the code length and the size of the binary [24]. The higher value of *x*, the more optimizations are performed during the last stage of compilation. It should be noted that, according to the GCC manual, –O3 may affect computation results and generate a binary larger than –O2, due to e.g., loops unrolling. Therefore, –O2 is usually recommended for release building profiles.

### 5.4. Enabling Hardware FPU Unit, Using CMSIS DSP Library

Since the release of the ARM Cortex-M4 core, STM32 microcontrollers have been equipped with IEEE 754 compliant hardware floating-point units. Depending on the model of the microcontroller, single- or double-precision units are available [23,25], supporting hardware accelerated operations on float32_t or float64_t types, respectively. The unit is disabled by default and had to be configured first.

One may also find it helpful to enable double promotion warnings (-Wdouble-promotion in GCC), to eliminate automatic casting of numbers to higher precision. Moreover, for calculations on float32_t or fixed-point q31_t numbers, which were highly optimized by taking advantage of dedicated intrinsic and SIMD operations, the CMSIS DSP library for ARM cores was considered. This library contains implementations of several common DSP algorithms, from among which arm_conv_partial_f32 and arm_scale_f32 functions were used for discrete convolution and vector scaling operations, respectively. The overall performance vastly improved. However, the truncated precision led to significant accumulated error ($\Delta e_{k}=\frac{\left|y_{DP}\left(k\right)-y_{SP}\left(k\right)\right|}{{|y}_{DP}\left(k\right)|}100\%=\;28.3\%$ for the last computed output sample), disqualifying the model $H_{NIOPDT}$ in this form from practical application.

### 5.5. Other Approaches

**Figure 8:**
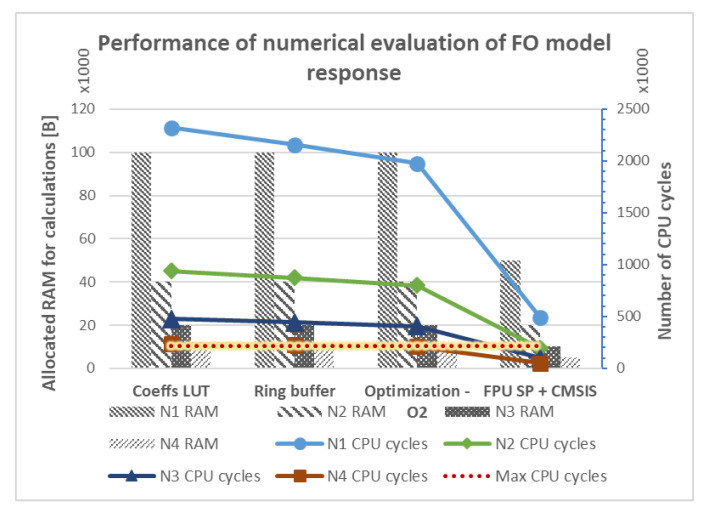
Software optimizations of the numerical algorithm for calculating output of model (3) with the maximum number of CPU cycles allowed (red dotted line).

Further optimizations are a topic of the ongoing research involving adaptive memory methods, parallel implementation of numerical algorithms and calculations using fixed-point arithmetic. Finally, assembly inlines placed in critical sections of the algorithms and platform-specific enhancements are being considered. However, these approaches are strictly platform-dependent and must be adopted for each architecture individually. The results of subsequent software optimizations are presented in Figure 8. The algorithm processing past $N_{4}=250$ samples, compiled with a –O2 flag, satisfied the initial timing requirement, calculating the output in a time shorter than the sampling period $t_{s}=1$ ms.

## 6. Conclusions

This study set out to address the problem of fractional-order model implementation. The non-local fractional differential GL operator involves a constantly growing number of calculations, which can be either bounded or replaced by an integer-order operator using one of the well-known approximation methods. A model of a UAV arm with a BLDC motor was implemented, using two approaches in the frequency- and time-domains: the Oustaloup approximation and numerical evaluation of the differential equation using the Grünwald–Letnikov definition with the Short Memory Principle. The performance of the algorithms was measured and compared. For orders $N_{ORA}>2$ of the Oustaloup approximation, similar step response characteristics were obtained. Moreover, in this case, the required buffer size was limited to only four feedforward and four feedback samples, vastly improving the calculation time and memory consumption. Higher accuracy could be obtained by different levels of reduction in the numbers of poles and zeros. Another approach, based on implementation of the Grünwald–Letnikov definition, required the introduction of the Short Memory Principle. In this case, programming optimization techniques allowed the computation time to be reduced by 15% or even 78% if CMSIS DSP and FPU hardware were used. This last result, however, required redesigning of the model. The methods described in this paper can be easily adapted and applied to other fractional-order models or control algorithms. Further work is underway, focusing on parallel implementation and optimization of fractional order numerical algorithms and designing a variable-, fractional-order PID controller with algorithms for determining the function of variable order.

## Figures and Tables

**Table 1:** Numerator and denominator coefficients of the discrete transfer functions (4) and (5), and of the continuous model (7).

Coeff	$H_{\mathrm{FOPDT}}\left(z\right)$	$H_{\mathrm{SOPDT}}\left(z\right)$	$H_{\mathrm{NIOPDT}}\left(z\right)$
$b_{0}$	1.123629474892 × 10^−4^	2.90801788577010 × 10^−6^	0.00141997809862250
$b_{1}$	1.901155870198 × 10^−3^	6.60568256821855 × 10^−6^	−0.00426750404873265
$a_{1}$	9.979752146549 × 10^−1^	1.993748864318110	2.99589971448314
$b_{2}$	-	0.25547387436067 × 10^−6^	0.00428081749129811
$a_{2}$	-	−0.993758633492438	−2.99180655152227
$b_{3}$	-	-	−0.00143328952852651
$a_{3}$	-	-	0.995906835027740
$T_{D}$	1228	1065	1000

**Table 2:** Accuracy of Short Memory Principle and of ORA with *balred* M=3.

	ISE	IAE	ITSE	ITAE	NRMSE	MaxAPE
$N_{0}=5000$	-	-	-	-	-	-
$N_{1}=2500$	$7.1496\times 10^{-7}$	$9.2601\times 10^{-4}$	$4.0238\times 10^{-6}$	$5.0596\times 10^{-3}$	99.91%	0.12%
$N_{2}=1000$	$5.7340\times 10^{-5}$	$1.1914\times 10^{-2}$	$2.9386\times 10^{-4}$	$5.8319\times 10^{-2}$	99.24%	0.65%
$N_{3}=500$	$2.8593\times 10^{-4}$	$2.9316\times 10^{-2}$	$1.3949\times 10^{-3}$	$1.3625\times 10^{-1}$	98.31%	1.25%
$N_{4}=250$	$3.9339\times 10^{-4}$	$3.4905\times 10^{-2}$	$1.9004\times 10^{-3}$	$1.6067\times 10^{-1}$	98.01%	1.43%
$N_{5}=100$	$1.5484\times 10^{-2}$	$2.4532\times 10^{-1}$	$6.6317\times 10^{-2}$	$1.0198\times 10^{0}$	87.53%	6.60%
$N_{6}=50$	$1.9433\times 10^{0}$	$2.6677\times 10^{0}$	$8.8778\times 10^{0}$	$1.1565\times 10^{1}$	0.00%	46.57%
$N_{ORA}=3$	$3.0352\times 10^{-6}$	$2.7942\times 10^{-3}$	$6.2302\times 10^{-6}$	$7.7747\times 10^{-3}$	99.83%	12.22%
$N_{ORA}=4$	$3.0566\times 10^{-6}$	$2.7666\times 10^{-3}$	$6.5758\times 10^{-6}$	$7.9428\times 10^{-3}$	99.82%	12.20%
$N_{ORA}=5$	$3.1344\times 10^{-6}$	$2.8097\times 10^{-3}$	$6.6456\times 10^{-6}$	$7.9841\times 10^{-3}$	99.82%	12.66%

## Abbreviations

The following abbreviations are used in this manuscript:

ADC	analog-to-digital converter
BLDC	brushless direct-current motor
CYCCNT	Data Watchpoint and Trace Cycle Count Register
(D)FOPDT	(discrete) first-order plus dead time
(D)NIOPDT	(discrete) non-integer-order plus dead time
(D)SOPDT	(discrete) second-order plus dead time
FIR	finite impulse response
FO	fractional-order
FOC	fractional-order calculus
FOTF	fractional-order transfer function
FPU	floating-point unit
GL	Grünwald-Letnikov
IAE	integral absolute error
IIR	infinite impulse response
IO	integer-order
ISE	integral square error
ISR	interrupt service routine
MaxAPE	maximum absolute percentage error
MCU	microcontroller unit
ORA	Oustaloup Recursive Approximation
SIMD	Single Instruction Multiple Data
SMP	Short Memory Principle
UAV	unmanned aerial vehicle
(V)FOPID	(variable) fractional-order proportional-integral-derivative controller
